# iHerd: an *i*ntegrative *h*i*e*rarchical graph *r*epresentation learning framework to quantify network changes and prioritize risk genes in *d*isease

**DOI:** 10.1371/journal.pcbi.1011444

**Published:** 2023-09-11

**Authors:** Ziheng Duan, Yi Dai, Ahyeon Hwang, Cheyu Lee, Kaichi Xie, Chutong Xiao, Min Xu, Matthew J. Girgenti, Jing Zhang

**Affiliations:** 1 Department of Computer Science, University of California, Irvine, California, United States of America; 2 Department of Computer Science, University of California, Davis, California, United States of America; 3 Department of Computational Biology, Carnegie Mellon University, Pittsburgh, Pennsylvania, United States of America; 4 Department of Psychiatry, School of Medicine, Yale University, New Haven, Connecticut, United States of America; 5 Clinical Neurosciences Division, National Center for PTSD, U.S. Department of Veterans Affairs, West Haven, Connecticut, United States of America; University of Michigan, UNITED STATES

## Abstract

Different genes form complex networks within cells to carry out critical cellular functions, while network alterations in this process can potentially introduce downstream transcriptome perturbations and phenotypic variations. Therefore, developing efficient and interpretable methods to quantify network changes and pinpoint driver genes across conditions is crucial. We propose a hierarchical graph representation learning method, called ***iHerd***. Given a set of networks, ***iHerd*** first hierarchically generates a series of coarsened sub-graphs in a data-driven manner, representing network modules at different resolutions (e.g., the level of signaling pathways). Then, it sequentially learns low-dimensional node representations at all hierarchical levels via efficient graph embedding. Lastly, ***iHerd*** projects separate gene embeddings onto the same latent space in its graph alignment module to calculate a rewiring index for driver gene prioritization. To demonstrate its effectiveness, we applied ***iHerd*** on a tumor-to-normal GRN rewiring analysis and cell-type-specific GCN analysis using single-cell multiome data of the brain. We showed that ***iHerd*** can effectively pinpoint novel and well-known risk genes in different diseases. Distinct from existing models, ***iHerd’s*** graph coarsening for hierarchical learning allows us to successfully classify network driver genes into early and late divergent genes (EDGs and LDGs), emphasizing genes with extensive network changes across and within signaling pathway levels. This unique approach for driver gene classification can provide us with deeper molecular insights. The code is freely available at https://github.com/aicb-ZhangLabs/iHerd. All other relevant data are within the manuscript and supporting information files.

## Author summary

In our study, we developed a new method called ***iHerd*** to better understand how genes work together within cells and how changes in these interactions can lead to various diseases. Our approach allows us to analyze complex gene networks and identify key genes responsible for network changes across different conditions. We applied ***iHerd*** to various data sets and found that it could effectively pinpoint genes linked to different diseases. Our method also helps classify these genes based on the extent of network changes they cause, providing deeper insights into the molecular mechanisms involved. Our research aims to improve our understanding of gene interactions and their role in disease development, ultimately contributing to the discovery of new therapeutic targets. The code is freely available for researchers to use and build upon in their work.

## Introduction

In biology, cells maintain highly coordinated gene expression patterns via precise spatiotemporal control to dictate essential molecular functions [1]. Numerous studies have reported that alterations in this dynamically controlled process (e.g., changes in gene regulation or gene co-expression relationships) can lead to expression-level perturbations, phenotypical changes, and a wide range of diseases [2]. Therefore, an important goal in systems biology has been to model such regulatory relationships and gene interactions as gene regulatory networks (GRNs) and gene co-expression networks (GCNs), respectively, using network representation analysis [3].

Recent advances in novel functional genomics and transcriptomic profiling assays have enabled direct analysis of gene regulation and interactions on a genome-wide scale, allowing us to construct high-confidence GRNs and GCNs across various biological conditions [4]. Moreover, the single-cell revolution, especially single-cell multi-omics sequencing, has expanded our understanding of such biological networks to the finest possible resolution–individual cells–providing an unprecedented opportunity to model and interpret network heterogeneity and dynamics in targeted cell types [5]. Lastly, transparent data-sharing initiatives from the scientific community have further provided scientists with direct access to population-scale functional genomic and single-cell sequencing data across diverse conditions [6]. Combined, these advances enable unprecedented opportunities for scientists to investigate transcription network dynamics and highlight novel risk genes for preventive medicine and drug development.

However, it remains computationally challenging to model network changes, i.e., rewiring events, for three reasons [7]. First, GRNs and GCNs are usually sparse and noisy, especially those inferred from single-cell omics data [8]. As a result, direct edge gain and loss counting methods are unable to precisely model and quantify network rewiring events. Second, multiple genes work together to carry out certain molecular functions (e.g., genes in the same signaling pathway), leaving them highly correlated and violating the independent and identically distributed assumptions [9]. Several models have been proposed to address such dependency using Latent Dirichlet Allocation [10]. While promising, those models still lack the ability to quantify network rewiring status within and across a particular pathway. Lastly, the availability of extensive genomics data provides rich features for genes, such as epigenetic status and mutation signatures [11]. Unfortunately, existing methods usually ignore node features in their network modeling and cannot provide hierarchical information [12]. Novel models are urgently needed to efficiently incorporate additional node features and offer gene pathway information in a hierarchical manner to facilitate accurate risk gene prioritization in disease studies.

**Fig 1:**
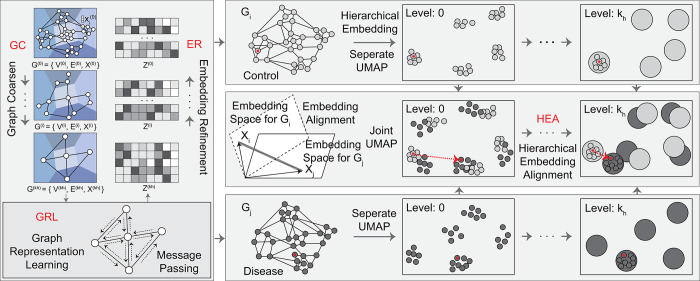
The overall framework of *iHerd*. It is an end-to-end learning framework, which contains a Graph Coarsen module, Graph Representation Learning module, Embedding Refinement module and Hierarchical Embedding Alignment module. The detail of each component is introduced in the Method Section.

To tackle these problems, we present a novel computational method, ***iHerd***, to efficiently quantify network rewiring status across different biological conditions. As shown in **Fig 1**, given a set of networks (e.g., GRNs and GCNs), ***iHerd*** first hierarchically generates a series of coarsened sub-graphs in a data-driven manner, representing gene communities at different resolutions to mimic signaling pathways. Then, it sequentially learns low-dimensional node representations at all hierarchical levels via efficient graph embedding. Lastly, ***iHerd*** includes a graph alignment module by projecting separate gene embeddings onto the same latent space to calculate a rewiring index for each gene at each hierarchical sub-graph level. This process makes it possible to measure network alterations and highlight genes with extensive network changes with direct interpretations. Distinct from existing methods, ***iHerd*** can directly extract complex gene dependencies from the observed networks and incorporate rich node features (e.g., transcriptomic, and epigenetic profiles) to jointly quantify network changes across conditions.

To demonstrate its effectiveness, we applied ***iHerd*** on a tumor-to-normal GRN rewiring analysis and cell-type-specific GCN analysis using single-cell multiome data in post-mortem brains. We showed that ***iHerd*** can effectively pinpoint novel and well-known risk genes in different disease models. Besides, ***iHerd***’s graph coarsening scheme for hierarchical learning allows us to successfully classify network driver genes into early and late divergent genes (EDGs and LDGs), representing genes with extensive network changes across and within signaling pathway levels. This unique approach for driver gene classification provides us with deeper molecular insights. We further validated the EDGs and LDGs via an independent transcriptomic analysis, demonstrating the power of our method.

In summary, ***iHerd*** can robustly capture rich and concise network topological information at multiple levels, hierarchically quantify network changes, and pinpoint network rewiring driver genes with molecular interpretations. We have implemented ***iHerd*** as a free software package available for the community to quantify network changes and prioritize risk genes in disease. With the explosion of available population-scale functional genomic and single-cell sequencing data across diverse conditions, we believe that ***iHerd*** will be a powerful tool for future studies.

## Results

### *iHerd* recovers key tumor-to-normal regulatory hierarchy changes by integrating large-scale ChIP-seq data

Transcription is synergistically regulated via cooperative interactions of different transcription factors (TFs). Lines of evidence indicate that the TF-TF regulatory network, a specific kind of GRN, naturally forms a hierarchy with a proclivity for downward information flow [13]. To some degree, scientists have argued that hierarchy, rather than connectivity, better reflects the importance of regulators, and perturbations of such hierarchical relationships can lead to a cascade of gene expression-level perturbations, causing a wide range of diseases. Therefore, we first used ***iHerd*** to analyze TF-TF GRN alterations in the process of oncogenesis.

Similar to previous work [10], we selected two widely used tier-1 ENCODE cell types K562 and GM12878 to roughly represent similar tumor-to-normal comparisons. Specifically, we compiled a shared TF-to-TF GRN by pruning the official ENCODE TF network constructed from ChIP-seq data (details in Method Section). As a result, we used two normal and tumor TF-to-TF GRNs as input for ***iHerd***, both with 86 edges from 40 common TFs.

**Fig 2:**
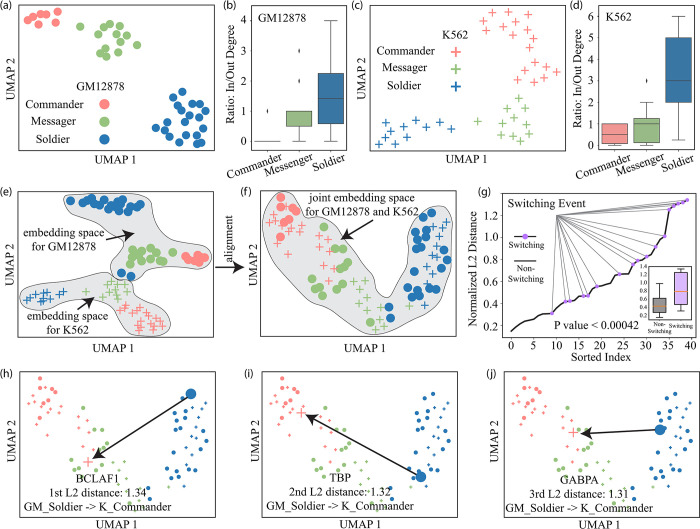
*iHerd* recovers the hierarchy change within the transcript factor (TF) GRNs. (a) The UMAP of embeddings for TFs in GM12878. (b) The boxplot of three clusters in GM12878 for the ratio of in degree and out degree. (c) The UMAP of embeddings for TFs in K562. (d) The boxplot of three clusters in K562 for the ratio of in degree and out degree. (e) The UMAP of embeddings for TF2 in GM12878 and K562 without the embedding alignment. (f) The UMAP of embeddings for TF2 in GM12878 and K562 after the embedding alignment. (g) The line plot of the sorted normalized L2 distance. The purple dot indicates there is a switching event for this TF. The boxplot of normalized l2 distance between non-switching TFs and switching TFs also demonstrates that the TF with a higher l2 distance has more chance to switch its cluster from GM12878 to K562. (h-j) The UMAPs of three TF examples switch their cluster from GM12878 to K562.

To better capture the TF hierarchy information, we specifically designed ***iHerd****’s* graph embedding method to mainly capture the topological identity information (see details in Methods Section). During the training, we first separately fitted our model on K562 and GM12878 TF-to-TF GRNs to obtain a latent representation for each TF. As shown in **Fig 2A–2D**, ***iHerd*** organized the 40 TFs into three distinct clusters with noticeable differences in their in/out degree ratios (0.57, 0.92, and 3.18 for K562). This pattern was highly consistent within the two cell types (0.14, 0.91, and 1.78 for GM12878). According to the in/out degree ratios, we analogized these three TF groups as commanders (in < out), messengers (in ≈ out), and soldiers (in > out) (**Fig 2A–2D**). Our findings are highly consistent with previous reports of network hierarchies in similar cell types.

Next, we aimed to quantify TF-to-TF GRN rewiring and highlight key TFs that change network hierarchies in the normal-to-tumor transition. Several methods have been developed for such analyses, such as the hierarchical score maximization [13], breadth-first-search [14], and vertex sort [15] algorithms. However, with only fixed and discrete TF hierarchy assignments, these methods fail to provide quantitative measures with sufficient resolution for precisely measuring GRN topological changes for each TF across conditions. In contrast, ***iHerd*** takes a different approach by aligning two graph embeddings onto the same latent space, where TFs are organized into distinct clusters by their topological roles (**Fig 2F**) rather than by their cell types (**Fig 2E**). Then, we can naturally calculate the normalized L2 distance ($d_{l2}^{norm}$) of the TF embeddings in the aligned latent space as the TF rewiring score. As a validation, we separated TFs into hierarchy-changing and hierarchy-preserving groups (purple and gray dots in **Fig 2F**) and compared their $d_{l2}^{norm}$ values in tumor and normal cells. As expected, hierarchy-changing TFs showed significantly higher $d_{l2}^{norm}$ values in the K562-GM12878 comparisons (0.78. vs. 0.42, P value 4E-4), validating the feasibility of our ***iHerd*** algorithm.

Lastly, we selected the top TFs with the largest $d_{l2}^{norm}$ in the tumor-to-normal GRNs and directly visualized their topological role changes in the uniform manifold approximation and projection (UMAP; enlarged points, **Fig 2H–2J**). For instance, *BCLAF1* demonstrated the largest $d_{l2}^{norm}$ in the normal-to-tumor transition by jumping from the soldier group in GM12878 to the commander group in K562. Interestingly, this finding is consistent with *BCLAF1*’s well-known role as a death-promoting TF with significantly upregulated gene expression in many cancer types [16]. Similarly, *TATA-binding protein* is upregulated by oncogenic signaling pathways and is suggested to be a critical component in the dysregulated signaling that occurs downstream of tumor-causing genetic lesions [17]. This TF also had a role change from the soldier group in GM12878 to the commander group in K562, as shown in **Fig 2I**. In addition, ***iHerd*** highlighted *GABPA*, a TF selectively recruited to the mutant form of the TERT promoter to activate TERT’s transcription in most human cancers [18]. All these TFs show hierarchical changes from normal to disease states and have known roles in cancer, indicating that ***iHerd*** can faithfully recover the changes of GRNs and quantify such network rewiring events across conditions with good interpretability.

### *iHerd* outperforms four baseline methods in the TF prioritization task

#### a) Simulation experiment settings

**Fig 3:**
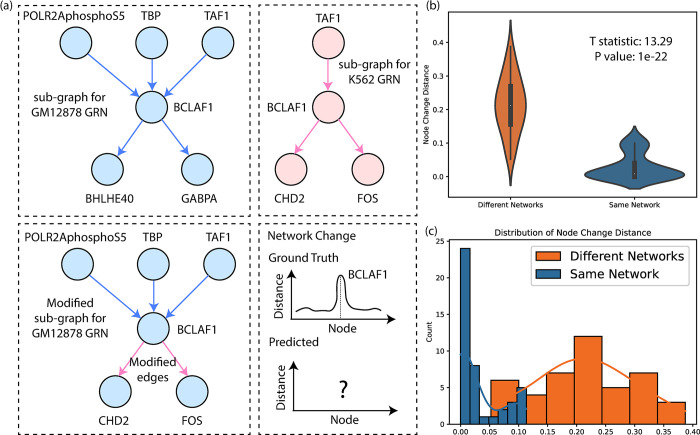
Simulated GRN experiments. (a) Simulation scheme on GRNs. (b) The violin plot of the false positive test. (c) The distributions of the node change distance for the false positive test.

Due to the lack of ground truth, we manipulated the GM12878 & K562 GRNs, as shown in **Fig 3A**. For node *i* in the GM12878 GRN ($G^{g_{0}}$), we simulated a new GRN ($G_{s}^{g_{i}}$) by randomly deleting its existing edges (e.g., BCLAF1-to-BHLHE40 and BCLAF1-to-GABPA, **Fig 3A**) and adding new edges from edges (BCLAF1-to-CHD2 and BCLAF1-to-FOS, **Fig 3A**) in the K562 GRN ($G^{k_{0}}$). We will not modify any edges in node i’s non-connecting nodes. Therefore, when comparing $G^{g_{0}}$ to $G_{s}^{g_{i}}$, the distance for node *i* (*d*_*i*_) should be larger than any *d*_*j*_, *j* ≠ *i* because there are almost no changes for node *j*. Similarly, we repeated this process for every TF in $G^{k_{0}}$. Note that one simulation would be discarded if this process gave rise to isolated nodes, or there is no difference in the two GRNs. In the end, we have 44 simulated network pairs.

#### b) Baseline methods

We benchmarked with four baseline methods (**Table 1** below).

**Table 1:** Summary of baseline methods.

Baseline Methods	Property	Limitations
Node Degree Change	Linear	Fails when in/out degrees remain constant despite edge changes
Clustering Coefficient Change	Non-linear	Fails when clustering coefficient remains constant despite edge changes
PageRank Change	Non-linear	Struggles to identify real node changes due to global scope.
Contrastive Learning	Non-linear	Fails to detect changes when node’s cluster membership remains the same.

- **Node Degree Change**. This baseline method ranks nodes by degree change in two directed GRNs. Degree change (*ΔD*) is the absolute sum of in-degree (*d*_*in*_) and out-degree (*d*_*out*_) changes. Given two graphs *G*_1_ and *G*_2_, each with *N* nodes, $\Delta D=|d_{in}(G_{1})-d_{in}(G_{2})|+|d_{out}(G_{1})-d_{out}(G_{2})|$.
- **Clustering Coefficient Change.** The clustering coefficient, *C*_*v*_, for a node *v* with *k*_*v*_ neighbors, reflects neighbor closeness. It’s computed as $C_{v}=\frac{2e_{v}}{k_{v}(k_{v}-1)}$, where *e*_*v*_ is the number of edges between the *k*_*v*_ neighbors. The absolute difference in clustering coefficients between the same nodes in two GRNs *G*_1_ and *G*_2_ is calculated as *ΔC* = |*C*(*G*_1_)−*C*(*G*_2_)|. Significant changes imply network connection changes.
- **PageRank Change.** The PageRank (*PR*) of a node *v* is given by $PR(v)=\frac{1-d}{N}+d*\Sigma_{(v,u)\in E}(\frac{PR(u)}{L(u)})$, where *d* is a damping factor (typically 0.85), *N* is the total number of nodes in the network, *E* is the set of edges in the network, *L*(*u*) is the number of outgoing links for a node *u*. After random initialization, PageRank values iteratively update until convergence. PageRank change between two GRNs *G*_1_ and *G*_2_, each with *N* nodes, is calculated as *ΔPR* = |*PR*(*G*_1_)−*PR*(*G*_2_)|.
- **Contrastive Learning.** This method applies the Louvain algorithm for initial clustering and optimizes node embeddings (*E*∈ℝ^*N*×*d*^, *N*: the number of nodes, *d*: embedding dimension) by adjusting inter/intra-cluster distances. The distances between node embeddings from two GRNs *G*_1_ and *G*_2_ are calculated as *ΔE* = ‖(*G*_1_)−*E*(*G*_2_)‖_2_, where ‖*‖_2_ represents the L-2 norm. Larger distances indicate significant connection changes.

#### c) Benchmarking results

We use the *R*@*n* (the chance that the modified TF was found within the top *n* predicted TFs) for benchmarking. Specifically, Let *x*_*i*_ be the changed node and *P*_*i*,*n*_ denotes the top *n* nodes predictions according to node distances, we can define *C*_*i*_ as a binary indicator function, where *C*_*i*_ = 1, if *x*_*i*_ is in *P*_*i*,*n*_ else 0. Then, *R*@*n* is defined as follows: $R@n=\frac{1}{\left|S\right|}\sum_{i}^{\left|S\right|}C_{i}$, where |*S*| is the total network pairs. As shown in **Table 2**, we found that iHerd consistently outperforms all other methods across all different levels of recall.

**Table 2:** TF prioritization benchmarking.

Methods	R@1	R@2	R@3	R@4	R@5
Node Degree	0.068	0.091	0.114	0.159	0.182
Clustering Coefficient	0.045	0.136	0.205	0.341	0.432
PageRank	0.205	0.295	**0.364**	0.409	0.432
Contrastive Learning	0.008	0.030	0.068	0.083	0.121
iHerd	**0.250**	**0.318**	**0.364**	**0.432**	**0.545**

#### d) False positive rate analysis

As suggested, we conducted a false positive rate analysis and examined two scenarios as shown in **Table 3**.

**Table 3:** False Positive Rate Analysis.

Two Scenarios	Input GRNs	Average Node Distance
Different Networks	$G^{g_{0}}\&G_{s}^{g_{i}}$	*Δd*_*f*_
Same Networks	$G_{s}^{g_{i}}\&G_{s}^{g_{i}}$	*Δd*_*b*_

- First, we calculated the average node distance (*Δd*_*f*_, foreground) by comparing different GRNs ($G^{g_{0}}\&G_{s}^{g_{i}}$, see “1.1. Simulation Experiment Settings” for details).
- Second, we calculated the average node distance (*Δd*_*b*_, background) by comparing identical GRNs ($G_{s}^{g_{i}}\&G_{s}^{g_{i}}$).

Both scenarios were tested across 44 modified versions of $G_{s}^{g_{i}}$. As shown in **Fig 3B** and **3C**, ***Δd***_***f***_ was significantly larger than ***Δd***_***b***_ (0.21 vs. 0.03, P = 1e-22 for two-sided t-test, demonstrating iHerd’s robustness.

### *iHerd* identifies early and late divergent genes across different cell types from single-cell RNA-sequencing data in post-mortem brains

Within a cell, a group of molecules works together via a cascade of biochemical reactions to control critical cellular functions, such as cell division or cell death ([19]). Alterations in such collaborative activities (i.e., “co-expression changes” in GCNs) can potentially introduce downstream transcriptome perturbations, phenotypic variations, and cell fate decisions [20]. Therefore, we next applied ***iHerd*** on cell-type-specific GCNs derived from single-cell data to investigate gene co-expression changes across different biological conditions.

**Fig 4:**
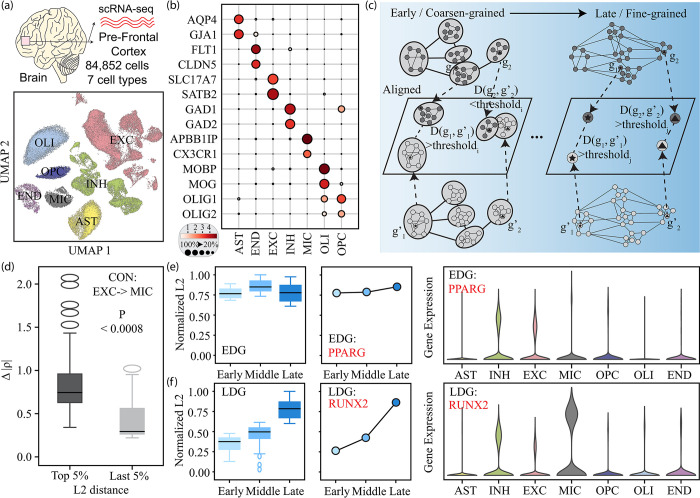
*iHerd* identifies different divergent genes between cell types. (a) Different neuronal and non-neuronal groups in UMAP using RNA. Here are seven samples and 84,852 cells. (b) The dot plot colored by gene expression for different cell types. These genes are differentially expressed across seven cell types. (c) The illustration of the discovery of different divergent genes. The L2 distance between g_2_ and g’_2_ is lower than the threshold at the early stage but exceeds the threshold at the late stage. While the L2 distance between g_1_ and g’_1_ exceeds the threshold for all stages. (d) The boxplot of normalized abstract correlation changes from excitatory neurons to macroglia between the top rewiring genes and the top conserved genes. Here we select 5% of genes with the largest L2 distance as the top rewiring genes and 5% of genes with the smallest l2 distance as the most conserved genes. The normalized L2 distance at different stages for EDG. We select one example EDG: PPARG, and its violin plot of gene expression indicates that PPARG is highly expressed in neurons. (f) The normalized L2 distance at different stages for LDG. We select one example LDG: RUNX2, and its violin plot of gene expression indicates that RUNX2 is highly expressed in microglia. "Middle" in iHerd refers to the first coarsening stage of the original network. With "Early", "Middle", and "Late" representing the coarsest, semi-coarse, and original networks, we can have deeper insights into gene behavior in different biological contexts.

To construct high-confidence GCNs, we generated single-cell-multiome data from seven post-mortem brain samples. After uniform pre-processing and strict quality control, 84,852 cells were kept and clustered into seven major cell types using canonical marker genes (**Fig 4A**, details in Methods Section; dot plot of marker genes is shown in **Fig 4B**). To overcome the sparsity of the single-cell sequencing data, we built the meta cells within each cluster and ran WGCNA [21] to construct high-confidence GCNs in a cell-type-specific manner.

First, we focused on the three normal control samples to investigate GCN changes across major brain cell types. Specifically, we kept 435 common highly variable genes in both excitatory neurons and microglia and constructed cell-type-specific GCNs as input for ***iHerd***. We enabled a graph coarsening process in our model for two purposes: 1) to distinguish gene interaction pattern changes within and across pathways; 2) to accelerate the training process on large graphs. As shown in **Fig 4C**, ***iHerd*** hierarchically learned node (gene) embeddings at each sub-graph level and then aligned these embeddings from different cell types to the same latent space. The $d_{l2}^{norm}$ from each level can be calculated accordingly, with the coarsest graph (early stage, light blue in **Fig 4C**) representing different pathway information and the full graph (late stage, dark blue in **Fig 4C**) capturing local community information. Then, we classified rewiring driver genes into EDGs and LDGs according to the $d_{l2}^{norm}$ patterns in each graph level. Specifically, EDGs are defined as genes with consistently high $d_{l2}^{norm}$ values across all graph levels, while LDGs are those with small $d_{l2}^{norm}$ values at coarse graph levels (light blue, **Fig 4C**) and high $d_{l2}^{norm}$ levels at dense graph levels (dark blue, **Fig 4C**).

Then, we selected 21 genes with the top 5% L2 distance between excitatory neurons and microglia at any graph level as candidate genes driving GCN changes across cell types. To validate these genes, we compared the sum of the correlation changes (∑Δ|*ρ*|) of our prioritized group with control genes (bottom L2 distance). Consistent with our predictions, the top rewiring genes showed larger correlation changes as compared to the bottom rewiring genes (0.72 vs. 0.26, P value 8E-4, **Fig 4D**), validating our prioritization scheme.

To further investigate EDGs and LDGs, we plotted an example of EDG and LDG in the UMAP for direct comparison (*PPARG* in **Fig 4E** and *RUNX2* in **Fig 4F,** respectively). Specifically, our highlighted EDG *PPARG* is a neuron-specific gene with low expression in microglia [22]. We found that *PPARG* showed large $d_{l2}^{norm}$ values from the coarsest graph to the full graph, as reflected by its consistently high rewiring scores. The hierarchical analysis indicated that *PPARG* jumped out of its original gene communities (e.g., pathways) with noticeable co-expression pattern changes. On the contrary, our highlighted LDG, *RUNX2*, is highly expressed in microglia but slightly downregulated in excitatory neurons [23]. *RUNX2* has an increasing pattern of $d_{l2}^{norm}$ values, with more significant $d_{l2}^{norm}$ values in refined, dense graphs, implying that it mainly changes co-expression relationships locally within the same gene community (e.g., pathways) without dramatic changes across different communities.

### *iHerd* highlights cell-type-specific risk genes in brain disorders

**Fig 5:**
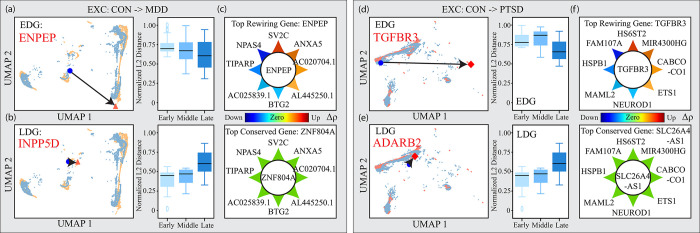
*iHerd* highlights extensive cell-type-specific divergent genes in brain disorders. (a-c) The analysis of excitatory neurons from control to MDD. (a) The UMAP of one example of an early divergent gene: ENPEP and the normalized L2 distance among different stages for EDG. (b) The UMAP of one example of a late divergent gene: INPP5D and the normalized L2 distance among different stages for LDG. (c) The normalized correlation changes for the top rewiring gene and the top conserved gene. The top rewiring gene “ENPEP” shows larger correlation changes with other genes while the top conserved gene “ZNF804A” almost has no correlation changes with other genes. (d-f) The analysis of excitatory neurons from control to PTSD. (d) The UMAP of one example of an early divergent gene: TGFBR3 and the normalized L2 distance among different stages for ED(e) The UMAP of one example of a late divergent gene: ADARB2 and the normalized L2 distance among different stages for LDG. (f) The normalized correlation changes for the top rewiring gene and the top conserved gene. The top wiring gene “TGFBR3” shows larger correlation changes with other genes while the top conserved gene “SLC26A4-AS1” almost has no correlation changes with other genes.

Lastly, we further explored GCN changes across different conditions to identify cell-type-specific risk genes for major depressive disorder (MDD) and post-traumatic stress disorder (PTSD) (**Fig 5**). We first performed a linkage disequilibrium score regression (LDSC) analysis on the single-cell ATAC-sequencing modality peaks for genome-wide association study summary statistics to highlight the most relevant cell types. Consistent with previous results, excitatory neurons showed the highest LDSC scores, indicating a strong disease association. Therefore, we focused on excitatory neurons to pinpoint disease driver genes.

***iHerd*** prioritized four genes in excitatory neurons: *ENPEP* and *INPP5D* for MDD and *TGFBR3* and *ADARB2* for PTSD (**Fig 5**). *ENPEP* was the highest-ranked EDG with consistently high $d_{l2}^{norm}$ scores at all graph levels (**Fig 5A**). Reports [24] indicate that *ENPEP* is associated with an inflammatory or immune response in MDD patients. *ENPEP* is involved in the negative regulation of transcription and nucleic acid metabolism, which contributes to MDD. We performed differential gene expression analysis on matched single-cell RNA-sequencing data and found that *ENPEP* is downregulated in excitatory neurons of MDD samples (0.662 log-fold change, P value 4.01E-44). Similarly, reports suggest that *INPP5D* is part of a significant KEGG pathway for phosphatidylinositol signaling in MDD [25]. ***iHerd*** highlighted *INPP5D* as the most significantly rewired LDG with extensive GCN rewiring within the same gene community (**Fig 5B**). Similarly in the PTSD-control comparison, the top EDG is *TGFBR3* (**Fig 5D**), which has been identified as a significantly modulated gene in stressful life event exposures [26]. Finally, researchers have demonstrated that *ADARB2* inhibits the activity of the other members of this gene family, suggesting that it plays a regulatory role in RNA editing for trauma-exposed individuals [27]. Consistently, we highlighted *ADARB2* (**Fig 5E**) as the most significant LDG, with significantly upregulated gene expression patterns in excitatory neurons from PTSD samples (0.809822 log-fold change, P value 2.82E-23). Combined, results demonstrate ***iHerd***’s ability to highlight extensive cell-type-specific divergent genes in brain disorders with molecular insights.

To validate the effectiveness of the selection of cell-type-specific risk genes by ***iHerd***, we examine the L2 distance in the common space across different conditions. **Fig 5C** lists the top rewiring gene *ENPEP* and top conserved gene *ZNF804A* from controls to MDD in excitatory neurons; **Fig 5F** lists the top rewiring gene *TGFBR3* and top conserved gene *SLC26A4-AS1* from controls to PTSD in excitatory neurons. Arrows around the circles point to other genes, and the colors of the arrows indicate normalized correlation changes. Between two genes, red denotes an increasing correlation while blue denotes a decreasing correlation, with deeper colors signifying larger changes. In the color bar shown, the green color indicates almost no correlation change. The top changed gene prioritized by ***iHerd*** shows large correlation changes with others while the top conserved gene shows almost no change, indicating that embeddings generated by ***iHerd*** reliably preserve the network correlation information. By faithfully reflecting the GCN changes, ***iHerd*** provides cell-type-specific risk genes with good interpretability.

### iHerd outperforms four baseline methods in the LDG/EDG distinction task

#### a) Simulation experiment settings

**Fig 6:**
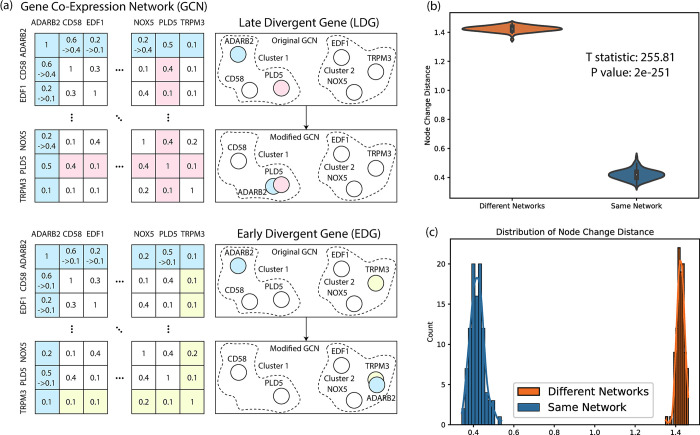
Simulated GCN experiments. (a) Simulation scheme on GCNs. (b) The violin plot of the false positive test. (c) The distributions of the node change distance for the false positive test.

We established artificial gene co-expression patterns for late and early divergent genes (LDGs and EDGs) using excitatory neurons’ GCN, as shown in **Fig 6A**.

- Construction of Late Divergent Gene (LDG). One gene in the GCN was designated a "modified gene," with a "template gene" chosen from the same cluster. We matched edge weights between the modified and template genes, forming a modified GCN with LDG.
- Construction of Early Divergent Gene (EDG). One gene in the GCN was designated a "modified gene," with a "template gene" chosen from a different cluster. We matched edge weights between the modified and template genes, forming a modified GCN with EDG.
- Repeat. Ultimately, we produced 2871 sample sets. Each set contained an original GCN, two modified GCNs (LDG and EDG), and a corresponding modified gene. Genes identified as individual clusters by the Louvain algorithm were excluded from this process.

#### b) Evaluation metric and results

We compared the ’modified gene’ changes across LDG and EDG scenarios. The proportion of simulations where EDG changes exceeded LDG changes, was used as the final accuracy measure. We found that iHerd consistently out-performed four baseline methods in accurately classifying 91.8% of cases (**Table 4**).

**Table 4:** EDG vs LDG benchmarking.

Methods	Accuracy
Node Degree	0.672
Clustering Coefficient	0.743
PageRank	0.529
Contrastive Learning	0.856
iHerd	**0.918**

#### c) False positive rate analysis

We carried out a false positive rate analysis on GCN data to evaluate the robustness of iHerd. As discussed in section 1.4, we computed the network distance, in two scenarios: 1) inputting two different GCNs (*Δd*_*f*_), and 2) repeating input of the same GCN (*Δd*_*b*_). We ran a control GCN through iHerd 100 times, comparing each result to a different GCN and calculated the average node distance among the control runs. As before, the background distance *Δd*_*b*_ (0.41) was significantly smaller than the foreground distance *Δd*_*f*_ (1.42, P value = 2e-251), confirming iHerd’s robustness (**Fig 6B** and **6C**).

### iHerd shows the robustness and biological relevance of its graph coarsen module

**Fig 7:**
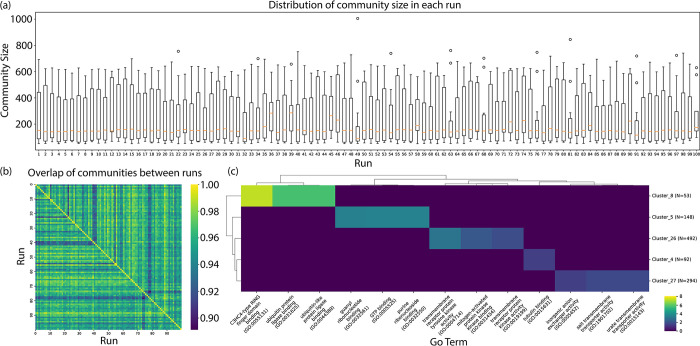
The robustness and biological relevance analysis of the graph coarsen module in *iHerd*. (a) The boxplot depicting the distribution of community sizes (node counts) in each run of iHerd. (b) A heatmap visualization of the overlap ratios calculated between communities identified across multiple runs of iHerd. (c) Enrichment heatmap from GO enrichment analysis of communities identified by iHerd.

We conducted additional experiments to evaluate the stability and reproducibility of iHerd on the same network (run 100 times). For each run, we calculated the size of the communities detected by the algorithm (focusing on communities with more than 50 nodes). We found the distribution of community sizes to be consistent across different runs (**Fig 7A**). Next, we repeated 100 runs to perform the Kolmogorov-Smirnov (KS) test and found the community size distribution between any run pair is similar (average P = 0.816). Additionally, we computed the overlap between communities from each pair of runs by focusing on one community. The heatmap in **Fig 7B** showed that most overlaps are above 0.9, indicating a high degree of consistency. Taken together, these results suggest that our algorithm shows strong consistency and stability across different runs, thereby providing reliable and reproducible outcomes. Next, we conducted an enrichment analysis using established GO Molecular Function terms to evaluate the biological relevance of the identified gene communities. After transforming the P-values to a "-log10" scale, we created a heatmap (**Fig 7C**) for visual comparison. The heatmap revealed unique biological processes within each community, each represented by different enriched GO terms. This diversity confirms iHerd’s effectiveness in identifying biologically significant and diverse gene communities."

### Parameter tuning for *iHerd*

**Fig 8:**
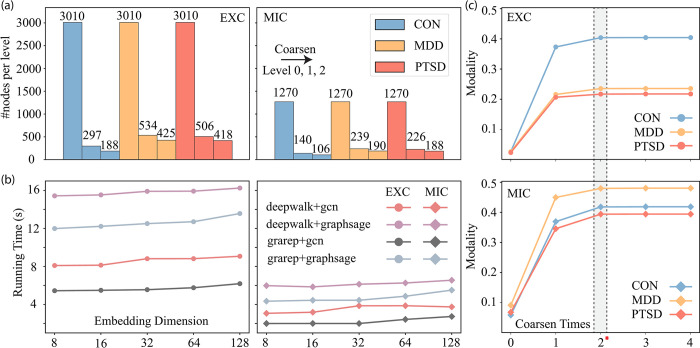
The parameter tuning for *iHerd*. (a) The bar plot of the number of nodes per level for controls and disease samples under excitatory neurons and microglia. (b) The line plot of running time with different embedding dimensions and different learning frameworks for controls under excitatory neurons and microglia. (c) The line plot of network modality with different coarsen times (zero coarsen times indicates the initial state).

We next analyzed the parameter tuning process of ***iHerd***. **Fig 8A** shows the number of genes at different levels for different conditions in excitatory neurons and microglia. Through the graph coarsening module, ***iHerd*** significantly reduces the number of nodes, thus providing node embeddings with richer global information. **Fig 8B** records the running time with respect to the embedding dimensions with different basic graph learning and refinement methods in controls in excitatory neurons and microglia. While a larger embedding dimension will slightly increase the running time, ***iHerd*** can generate embeddings for all hierarchical levels within 20 seconds without a graphics processing unit, showing excellent scalability. **Fig 8C** records the modality changes as the coarsening times increase. For different conditions in excitatory neurons and microglia, the network modality barely grows after two graph coarsening operations. Based on this result, we chose two coarsening times as the final hyper-parameters to achieve better hierarchical embeddings.

## Discussion

This paper presents a computational method, called ***iHerd***, to quantify network changes and prioritize risk genes in disease. While recent developments have shed light on gene prioritization in diseases, it is still hard for people to quantify changes in GRNs and GCNs. Leveraging the advantage of hierarchical graph neural networks, ***iHerd*** coarsens the fine-grained input network to a coarsen-grained network, learns the embeddings for each cluster, and refines the embedding for each node. By aligning embeddings of different networks, we can quantitatively analyze changes in different genes at different levels. To prove the effectiveness of ***iHerd***, we analyze the network changes on GRNs and GCNs. We showed that ***iHerd*** can faithfully highlight the changed TFs and the divergent genes in each respective network across different cell types and conditions. We introduced two kinds of divergent genes here: early divergent gene and late divergent gene, providing a more insightful gene prioritization analysis and enhancing the interpretability of our model.

It is worth mentioning that ***iHerd*** is a flexible framework and it can combine different graph learning methods (focus on structural information or local neighborhood) depending on the input network. Meanwhile, how to effectively combine the node features into this framework would be a promising direction of future exploration. Finally, we have implemented ***iHerd*** as an open-source software that is freely downloadable to the public. With the exponential growth of disease data, ***iHerd*** can be a useful tool for the community to prioritize genes in diseases and quantify their changes at different stages.

## Methods

As shown in **Fig 1**, ***iHerd*** has four main components: Graph Coarsen module (GC), Graph Representation Learning module (GRL), Embedding Refinement module (ER), and the Hierarchical Embedding Alignment module (HEA). We will introduce the data preprocessing steps and each module of ***iHerd*** in the following sections.

### Data preprocessing

#### Gene regulatory networks data preprocessing

We obtained the ENCODE networks for two cell lines, GM12878 and K562, and focused on their respective TF regulatory networks, which are subnetworks of the official ENCODE networks (We used the 2017 freeze for all ChIP-seq data, and the detailed experiment ID has been included in S1 Table). The official ENCODE network for GM12878 consists of 8,633 genes (including 101 TFs), and 24,093 edges. Similarly, for the K562 cell line, the official ENCODE network consists of 11,250 genes (including 197 TFs), and 43,253 edges. We provided a detailed **Table 5** describing the network statistics. To investigate the regulation of TFs, we extracted these TFs from the original networks and focused on their interactions. This process resulted in two TF regulatory networks, each sharing a common set of 40 TFs. These TF-to-TF GRNs were used as inputs for further analysis in iHerd.

**Table 5:** Statistics of GRNs.

		GM12878	K562
TF-to-Gene	# of Nodes	8,633	11,250
	# of Edges	24,093	43,263
TF-to-TF	# of Nodes	69	151
	# of Edges	201	825
Common TF-to-TF	# of Nodes	40	
	# of Edges	89	

### scRNA-seq data preprocessing and quality control

A series of quality control steps were taken to reduce the effects of high dimensionality, low capture efficiency, and noise in scRNA-seq data. First, CellBender was used to eliminate technical artifacts and background noise in each scRNA-seq sample. Then DoubletDetection and Scrublet were used to remove doublets from each scRNA-seq sample. All samples were aggregated in Pegasus (v.1.5.0), a python tool for analyzing transcriptomes of single cells Then, cells were further filtered based on the following criteria: at most 10% mitochondrial genes, at most 2% ribosomal genes, at least 200 genes, and at least 500 UMIs. Mitochondrial, sexual, and ribosomal genes were excluded, and only the robust genes were included in the final data object. After all the filtration steps, dimensionality reduction, batch correction with Harmony, clustering, and annotation were performed in Pegasus. Seven brain PFC samples were used in our experiments: three CON samples (MS000800, MS0184LL, MS0198ZZ), two PTSD samples (MS0098CC, MS0146ZZ), and two MDD samples (MS0096AA, MS0204RR). We then constructed cell-type-specific gene co-expression networks based on the following steps. In the networks, we only considered protein-coding genes that are top 5,000 highly variable and are expressed in at least 5% of the cells, respectively in each cell type. First, to overcome the sparsity of scRNA-seq data, we constructed metacells in a procedure like scWGCNA [28]. Each metacell is an aggregation of 100 cells that are nearest neighbors in 20 PCA dimensions. We required that the aggregated cells come from the same cell type, same gender group, and the same disease condition. All metacells from the same cell type are then collected to form the metacell-by-gene expression matrices normalized by metacells and scaled by genes. Finally, for each condition and each cell type, we computed unsigned Pearson correlations for all pairs of genes to construct gene co-expression networks (GCN). Then GCNs with different conditions and cell types were fed into iHerd for further analysis. The scRNA-seq data was generated by the Girgenti lab, which can be accessed by GSE216270.

### Graph coarsen

Given a graph *G* = (*V*, *E*, *X*), *V* is a set of *n* nodes; *E* is a set of *m* edges and $X\in R^{n\times d_{x}}$ is the node features, where *d*_*x*_ is the dimension of node features. The Graph Coarsen module generates a series of hierarchical attributed networks from the finest to the most coarsened: ${G=G}^{0}≻\cdots ≻G^{i}≻\cdots ≻G^{k_{h}}$, where ≻ denotes the coarsen operation, *G*^*i*^ is the coarsened graph on the *i*-th coarsen layer and *k*_*h*_ is the predefined number of coarsen layers. In this module, the Louvain algorithm [29] is employed to detect non-overlapping communities and form super-nodes for coarser graphs. This module consists of two phases: partition and reconstruction.

### Partition

The partition phase aims to maximize modularity, which is a measure of the quality of divisions inside a network structure. For a community *c* in a graph, its modularity is defined as:

$$Q_{c}=\frac{1}{2m}\sum_{ij}\left[A_{ij}-\frac{k_{i}k_{j}}{2m}\right],$$ (1)

where *A*_*ij*_ denotes the edge weight between nodes *i* and *j* in community *c*, *m* is the sum of all edge weights, and *k*_*i*_ and *k*_*j*_ are the sums of edge weights of nodes *i* and *j* respectively. Given a graph *G*^*i*^ = (*V*^*i*^, *E*^*i*^, *X*^*i*^), the partition phase starts by assigning each node to its own community. For each vertex *v*_*x*_^*i*^∈*V*^*i*^, the change of modularity *ΔQ* is calculated by removing *v*_*x*_^*i*^ from its current community to its neighbors After *ΔQ* of all neighbor communities of *v*_*x*_^*i*^ have been calculated, *v*_*x*_^*i*^ is re-assigned to the community that results in the highest modularity gain. The partition phase repeats until a local maximum of modularity is reached. In our experience, however, we found that two iterations of this process typically lead to an optimal trade-off between modularity and computational efficiency. A more detailed overview of the parameter tuning process is shown in the “Parameter tuning for iHerd” section.

### Reconstruction

The reconstruction phase constructs a new coarser graph *G*^*i*+1^ = (*V*^*i*+1^, *E*^*i*+1^, *X*^*i*+1^) and in this process, each super-node (community) of graph *G*^*i*^ becomes a new node *v*^*i*+1^∈*V*^*i*+1^. An edge *e*_*pq*_^*i*+1^∈*E*^*i*+1^ is created if nodes *v*_*a*_^*i*^ and *v*_*b*_^*i*^ at a coarser level *i* has an edge *e*_*ab*_^*i*^∈*E*^*i*^, where *v*_*a*_^*i*^ and *v*_*b*_^*i*^ are assigned to super-nodes *v*_*p*_^*i*+1^ and *v*_*q*_^*i*+1^. Edge weight of *e*_*pq*_^*i*+1^ is updated as the summation of all edge weights between those two communities. Then, the new graph *G*^*i*+1^ with more concise topological proximity of the original network *G*^0^ is stored and passed to this module again. This GC module continues with the new coarsen graph *G*^*i*+1^ and repeats the process until *k*_*h*_ times or there is no obvious modularity gain. After this module, we get a series of networks at different coarseness levels: $G^{0}≻\cdots ≻G^{i}≻\cdots ≻G^{k_{h}}$ such that |*V*^*i*−1^|>|*V*^*i*^| and |*E*^*i*−1^|>|*E*^*i*^|, *i* = 1,2,3,…,*k*_*h*_.

### Embedding on the coarsest graph

After the GC module, an embedding of the network at the coarsest granularity is generated. The coarsest network $G^{k_{h}}=(V^{k_{h}},E^{k_{h}},X^{k_{h}})$, which maintains the most concise structural information of the original network, is passed into the graph representation learning module to produce the first proximate graph representation of the original network. The embedding of the coarsest hierarchical attributed network is generated by the following equation: $Z^{k_{h}}=GRL(G^{k_{h}})$, where GRL is the graph representation learning module and $Z^{k_{h}}\in R^{|V^{k_{h}}|\times d}$.

### Graph coarsen embedding refinement

The Embedding Refinement module (ER) leverages unsupervised graph neural network (GNN) to refine the coarsest embedding to the finest-grained embedding: $Z^{k_{h}}\to \cdots \to Z^{i}\to \cdots \to Z^{0}$. The primary goal of this module is to attain a trained GNN model that can produce the finer-grained embedding *Z*^*i*^ as equation: *Z*^*i*^ = *ER*(*Z*^*i*+1^, *G*^*i*^). Then, through the GNN model, *Z*^0^ for *G*^0^ can then be iteratively attained from the coarsest network $\left(Z^{k_{h}},G^{k_{h}}\right)$ to the finest grained one in a backpropagation manner while preserving the topological structure information of the coarsest network.

### Initialization

Before the GNN model is employed to generate *Z*^*i*^ from (*Z*^*i*+1^, *G*^*i*^), *Z*^*i*^ is initialized based on $\left(Z^{i+1},G^{i}):Z^{i}=Initialize(Z^{i+1},G^{i}).\;Initialize(\cdot \right)$ gives the node representations of *z*_*j*_^*i*+1^∈*Z*^*i*+1^ to *z*_*s*_^*i*^∈*Z*^*i*^, where *v*_*s*_^*i*^ is inside the super-node (community) *V*_*j*_^*i*+1^.

### Unsupervised graph neural network

When a trained GNN model is employed, the embedding of a finer network $Z^{i}\in R^{|V^{i}|\times d}$ is generated: *Z*^*i*^ = *H*(*Z*^*i*^, *G*^*i*^), where *H*(·) denotes a layer-wise linear GraphSAGE model [30]. A single layer is defined as *H*^(*l*)^(·), ∀*l*∈{1,…,*L*}, where *L* is the total number of layers in the model. For a given node *v*^*i*^∈*V*^*i*^, *H*^(*l*)^(*Z*^*i*^, *G*^*i*^) consists of the following two steps:

$${h^{\left(l\right)}}_{N(v)}\leftarrow AGGREGATE_{l}\left(\{{h_{u}}^{\left(l-1\right)},\forall u\in N(v)\}\right),$$ (2)

$${h^{\left(l\right)}}_{v}\leftarrow \sigma \left(W^{\left(l\right)}\cdot CONCAT({h_{v}}^{\left(l-1\right)},{h^{\left(l\right)}}_{N(v)})\right).$$ (3)

Here, {*h*_*u*_^(*l*−1)^, ∀*u*∈*N*(*v*)} denotes the representations of the neighbors of *v* in the last layer (*l*−1) and the neighbors from the neighborhood function *N*(*v*). *AGGREGATE*_*k*_(·) denotes a function that will aggregate the representations of neighbor nodes, such as the mean operator, LSTMs, max-pooling, and so on. *CONCAT*(*·*) is the concatenation operator of two embedding matrices. *W*^(*l*)^ is a layer-specific trainable weight matrix and *σ*(·) is a non-linear activation function [31]. At the final layer, we attain representation of each node at coarsen level *i*: *z*_*v*_^*i*^←*h*_*v*_^*L*^, ∀*v*∈*V*^*i*^. To train this GNN model and get trained *W*^(*l*)^ for *L* layers, we define a loss function as follows:

$$Loss=\frac{1}{\left|V^{k_{h}}\right|}\|Z^{k_{h}}-H^{\left(l\right)}(Z^{k_{h}},G^{k_{h}})\|.$$ (4)

The base embedding generated from the GRL module plays the part of “ground-truth” embedding of $G^{k_{h}}$. The difference between $Z^{k_{h}}$ and the predicted embedding $H^{\left(l\right)}(Z^{k_{h}},G^{k_{h}})$ is the training loss. The model is trained only with the coarsest graph $G^{k_{h}}$ once. Then it will be employed to generate graph representations for all previous levels as described above. The unsupervised graph neural network model in this module can also be flexible.

### Hierarchical embedding alignment

In the Graph Coarsen module, the hierarchical embedding of a coarser network contains more concise global clustering structural information at a higher level, while that of a finer network contains information at a more local level [32]. Since embeddings at different levels provide different characteristics, we need to compare embeddings at all levels to get an integrated insight into the network changes.

### Mapping matrices by solving the orthogonal proscrutes problem

Due to the randomness of initialization and the training process, we cannot directly compare the embeddings from two different running times [33]. We can calculate the distance between embeddings, by solving the orthogonal proscrutes problem [34] and mapping all embeddings onto a common space. Given two matrices *M*_1_∈*R*^*n*×*d*^, *M*_2_∈*R*^*n*×*d*^, we search for a unitary matrix *U*∈*R*^*d*×*d*^ that best maps *M*_2_ to *M*_1_, which formulated by minimizing ${\left\|M_{1}-M_{2}U^{2}\right\|}_{F}^{2}$, where ‖·‖_*F*_ is the Frobenius norm. In the HEA module, ***iHerd*** generates a mapping matrix *U*_(*s*1,*s*2)_ for the representations $Z_{s1}^{0}$ and $Z_{s2}^{0}$ at the finest granularity level between two samples *s*1 and *s*2. It is then used to align other embeddings at different coarsen levels for ${(Z}_{s1}^{i},\ldots ,Z_{s1}^{k_{h}})$ and $\left(Z_{s2}^{i},\ldots ,Z_{s2}^{k_{h}}\right)$.

### Distance calculation

For any pair of hierarchical embeddings at each level ${(Z}_{s1}^{i},Z_{s2}^{i}),i\in \{1,2,\ldots ,k_{h}\}$, distances are calculated for all nodes after the alignment. Here, we define two categories of genes that need to be prioritized: early divergent genes (EDGs) and late divergent genes (LDGs). EDGs maintain high distances at both coarsen-grained and fine-grained levels. They diverge at early stages, and consistently show their high divergence in later stages. In contrast, LDGs show high distances only at fine-grained levels. For LDGs, significant topological differences only occur on the local scale.

Let $z_{\left(v_{1}^{0},s1\right)}^{0}$ and $z_{\left(v_{1}^{0},s2\right)}^{0}$ denote the embedding vectors of node $v_{1}^{0}$ in samples *s*1 and *s*2 respectively at the finest-grained level. We also have $z_{\left(v_{1}^{k_{h}},s1\right)}^{k_{h}}$ and $z_{\left(v_{1}^{k_{h}},s2\right)}^{k_{h}}$, where $\left(v_{1}^{k_{h}},s1\right)$ and $\left(v_{1}^{k_{h}},s2\right)$ are the corresponding super-nodes (communities) for $v_{1}^{0}$ at the most coarsen-grained level *k*_*h*_ for samples *s*1 and *s*2 respectively. The filter equations for EDGs and LDGs are as follows:

$$D\left(z_{\left(v_{1}^{k_{h}},s1\right)}^{k_{h}},z_{\left(v_{1}^{k_{h}},s2\right)}^{k_{h}}\right)\approx D(z_{\left(v_{1}^{0},s1\right)}^{0},z_{\left(v_{1}^{0},s2\right)}^{0}),$$ (5)

$$D\left(z_{\left(v_{1}^{k_{h}},s1\right)}^{k_{h}},z_{\left(v_{1}^{k_{h}},s2\right)}^{k_{h}}\right)\leq D(z_{\left(v_{1}^{0},s1\right)}^{0},z_{\left(v_{1}^{0},s2\right)}^{0}),$$ (6)

where *D*(·) denotes a nested function for mapping embeddings into a common space and calculating distance values between nodes. A node that falls into either of these two categories should be prioritized and picked out for further analysis.

## Supporting information

S1 Table Details of the 2017 freeze for all ChIP-seq data. (CSV)

## References

[1] Goglia AG, Toettcher JE. A bright future: optogenetics to dissect the spatiotemporal control of cell behavior. Curr Opin Chem Biol. 2019;48:106–13. doi: 10.1016/j.cbpa.2018.11.010 30529586 PMC6382565

[2] Braenne I, Onengut-Gumuscu S, Chen RX, Manichaikul AW, Rich SS, Chen WM, et al. Dynamic changes in immune gene co-expression networks predict development of type 1 diabetes. Sci Rep-Uk. 2021;11(1). doi: 10.1038/s41598-021-01840-z 34811390 PMC8609030

[3] AbuQamar SF, El-Tarabily KA, Sham A. Co-expression Networks in Predicting Transcriptional Gene Regulation. Methods Mol Biol. 2021;2328:1–11. doi: 10.1007/978-1-0716-1534-8_1 34251616

[4] Kartha VK, Duarte FM, Hu Y, Ma S, Chew JG, Lareau CA, et al. Functional inference of gene regulation using single-cell multi-omics. Cell Genom. 2022;2(9). doi: 10.1016/j.xgen.2022.100166 36204155 PMC9534481

[5] Zhang L, Zhang J, Nie Q. DIRECT-NET: An efficient method to discover cis-regulatory elements and construct regulatory networks from single-cell multiomics data. Sci Adv. 2022;8(22):eabl7393. doi: 10.1126/sciadv.abl7393 35648859 PMC9159696

[6] Xiong L, Tian K, Li YZ, Ning WX, Gao X, Zhang QC. Online single-cell data integration through projecting heterogeneous datasets into a common cell-embedding space. Nature Communications. 2022;13(1). doi: 10.1038/s41467-022-33758-z 36253379 PMC9574176

[7] Hollander M, Do T, Will T, Helms V. Detecting Rewiring Events in Protein-Protein Interaction Networks Based on Transcriptomic Data. Front Bioinform. 2021;1:724297. doi: 10.3389/fbinf.2021.724297 36303788 PMC9581068

[8] Banf M, Rhee SY. Computational inference of gene regulatory networks: Approaches, limitations and opportunities. Bba-Gene Regul Mech. 2017;1860(1):41–52. doi: 10.1016/j.bbagrm.2016.09.003 27641093

[9] Song X, Jin P, Qin S, Chen L, Ma F. The evolution and origin of animal Toll-like receptor signaling pathway revealed by network-level molecular evolutionary analyses. PLoS One. 2012;7(12):e51657. doi: 10.1371/journal.pone.0051657 23236523 PMC3517549

[10] Zhang J, Lee D, Dhiman V, Jiang P, Xu J, McGillivray P, et al. An integrative ENCODE resource for cancer genomics. Nat Commun. 2020;11(1):3696. doi: 10.1038/s41467-020-14743-w 32728046 PMC7391744

[11] McLaren W, Gil L, Hunt SE, Riat HS, Ritchie GRS, Thormann A, et al. The Ensembl Variant Effect Predictor. Genome Biol. 2016;17. 27268795 10.1186/s13059-016-0974-4 PMC4893825

[12] Ying R, You JX, Morris C, Ren X, Hamilton WL, Leskovec J. Hierarchical Graph Representation Learning with Differentiable Pooling. Advances in Neural Information Processing Systems 31 (Nips 2018). 2018;31.

[13] Cheng C, Andrews E, Yan KK, Ung M, Wang DF, Gerstein M. An approach for determining and measuring network hierarchy applied to comparing the phosphorylome and the regulome. Genome Biol. 2015;16. 25880651 10.1186/s13059-015-0624-2 PMC4404648

[14] Yu HY, Gerstein M. Genomic analysis of the hierarchical structure of regulatory networks. P Natl Acad Sci USA. 2006;103(40):14724–31. doi: 10.1073/pnas.0508637103 17003135 PMC1595419

[15] Jothi R, Balaji S, Wuster A, Grochow JA, Gsponer J, Przytycka TM, et al. Genomic analysis reveals a tight link between transcription factor dynamics and regulatory network architecture. Mol Syst Biol. 2009;5. doi: 10.1038/msb.2009.52 19690563 PMC2736650

[16] Liu J, Li JY, Sun Z, Duan YM, Wang FQ, Wei GW, et al. Bcl-2-associated transcription factor 1 Ser290 phosphorylation mediates DNA damage response and regulates radiosensitivity in gastric cancer. J Transl Med. 2021;19(1). doi: 10.1186/s12967-021-03004-z 34372878 PMC8351323

[17] Johnson SAS, Dubeau L, Kawalek M, Dervan A, Schonthal AH, Dang CV, et al. Increased expression of TATA-binding protein, the central transcription factor, can contribute to oncogenesis. Mol Cell Biol. 2003;23(9):3043–51. doi: 10.1128/MCB.23.9.3043-3051.2003 12697807 PMC153209

[18] Bell RJA, Rube HT, Kreig A, Mancini A, Fouse SD, Nagarajan RP, et al. The transcription factor GABP selectively binds and activates the mutant TERT promoter in cancer. Science. 2015;348(6238):1036–9. 25977370 10.1126/science.aab0015 PMC4456397

[19] Zhang B, Horvath S. A general framework for weighted gene co-expression network analysis. Stat Appl Genet Mol. 2005;4. doi: 10.2202/1544-6115.1128 16646834

[20] Chen C, Cheng L, Grennan K, Pibiri F, Zhang C, Badner JA, et al. Two gene co-expression modules differentiate psychotics and controls. Mol Psychiatr. 2013;18(12):1308–14. doi: 10.1038/mp.2012.146 23147385 PMC4018461

[21] Langfelder P, Horvath S. WGCNA: an R package for weighted correlation network analysis. Bmc Bioinformatics. 2008;9. 19114008 10.1186/1471-2105-9-559 PMC2631488

[22] Gupta C, Xu J, Jin T, Khullar S, Liu X, Alatkar S, et al. Single-cell network biology characterizes cell type gene regulation for drug repurposing and phenotype prediction in Alzheimer’s disease. Plos Comput Biol. 2022;18(7):e1010287. doi: 10.1371/journal.pcbi.1010287 35849618 PMC9333448

[23] Gosselin D, Skola D, Coufal NG, Holtman IR, Schlachetzki JCM, Sajti E, et al. An environment-dependent transcriptional network specifies human microglia identity. Science. 2017;356(6344). doi: 10.1126/science.aal3222 28546318 PMC5858585

[24] Song GG, Kim JH, Lee YH. Genome-Wide Pathway Analysis in Major Depressive Disorder. J Mol Neurosci. 2013;51(2):428–36. doi: 10.1007/s12031-013-0047-z 23794217

[25] Kuan PF, Waszczuk MA, Kotov R, Marsit CJ, Guffanti G, Gonzalez A, et al. An epigenome-wide DNA methylation study of PTSD and depression in World Trade Center responders. Transl Psychiat. 2017;7. doi: 10.1038/tp.2017.130 28654093 PMC5537648

[26] Lopizzo N, Tosato S, Begni V, Tomassi S, Cattane N, Barcella M, et al. Transcriptomic analyses and leukocyte telomere length measurement in subjects exposed to severe recent stressful life events. Transl Psychiat. 2017;7. doi: 10.1038/tp.2017.5 28221367 PMC5438034

[27] Morey RA, Garrett ME, Stevens JS, Clarke EK, Haswell CC, van Rooij SJH, et al. Genetic predictors of hippocampal subfield volume in PTSD cases and trauma-exposed controls. Eur J Psychotraumato. 2020;11(1). doi: 10.1080/20008198.2020.1785994 33029326 PMC7473168

[28] Morabito S, Miyoshi E, Michael N, Shahin S, Martini AC, Head E, et al. Single-nucleus chromatin accessibility and transcriptomic characterization of Alzheimer’s disease. Nat Genet. 2021;53(8):1143–+. doi: 10.1038/s41588-021-00894-z 34239132 PMC8766217

[29] Blondel VD, Guillaume JL, Lambiotte R, Lefebvre E. Fast unfolding of communities in large networks. J Stat Mech-Theory E. 2008.

[30] Hamilton WL, Ying R, Leskovec J. Inductive Representation Learning on Large Graphs. Adv Neur In. 2017;30.

[31] Wang YY, Duan ZH, Liao BB, Wu F, Zhuang YT. Heterogeneous Attributed Network Embedding with Graph Convolutional Networks. Aaai Conf Artif Inte. 2019:10061–2.

[32] Duan Z, Xu H, Wang Y, Huang Y, Ren A, Xu Z, et al. Multivariate time-series classification with hierarchical variational graph pooling. Neural Netw. 2022;154:481–90. doi: 10.1016/j.neunet.2022.07.032 35970026

[33] Du L, Wang Y, Song GJ, Lu ZC, Wang JS. Dynamic Network Embedding: An Extended Approach for Skip-gram based Network Embedding. Proceedings of the Twenty-Seventh International Joint Conference on Artificial Intelligence. 2018:2086–92.

[34] H SP. A generalized solution of the orthogonal procrustes problem. Psychometrika. 1966;31(1):1–10.

